# Rapamycin attenuates acute lung injury induced by LPS through inhibition of Th17 cell proliferation in mice

**DOI:** 10.1038/srep20156

**Published:** 2016-02-18

**Authors:** Zhao Yan, Zhang Xiaoyu, Song Zhixin, Qi Di, Deng Xinyu, Xia Jing, He Jing, Deng Wang, Zhong Xi, Zhang Chunrong, Wang Daoxin

**Affiliations:** 1 Department of Pulmonary and Critical Care Medicine, the Second Affiliated Hospital of Chongqing Medical University, 76 Linjiang Road, Yuzhong District, Chongqing 400010, China; 2 Key Laboratory of Diagnostic Medicine Designated by the Ministry of Education, Chongqing Medical University, 1 Yixueyuan Road, Yuzhong District, Chongqing 400010, China; 3 Department of Emergency Medicine, the Yongchuan Affiliated Hospital of Chongqing Medical University, 439 Xuanhua Road, Yongchuan District, Chongqing 402160, China

## Abstract

Th17 cells have been confirmed to increase neutrophils through cytokine secretions. ALI/ARDS are characterized as neutrophil infiltration in inflammation cases; however, there is conflicting information concerning the role of Th17 cells in ALI/ARDS, as well as their potential treatment value. We measured Th17-linear cytokines in the plasma of patients with sepsis-related ARDS. The consistently high levels of IL-17 and IL-22 in the nonsurvivors suggested that overreaction of the Th17-mediated immune response may be a risk factor for poor outcomes. Th17 linear cytokines were also increased in an LPS-induced murine model of acute lung injury, along with neutrophil accumulation. The mice that completely lacked IL-17 failed to accumulate and activate neutrophils. Lung inflammation was obviously attenuated in the IL-17^−^/^−^ mice. Meanwhile, the neutrophil count was markedly increased in the healthy WT mice challenged with recombinant IL-22 and IL-17. Rapamycin attenuated lung injury by inhibiting the differentiation of Th17 cells through RORγt and STAT3 dysfunction. Furthermore, we demonstrated that SOCS3 and Gfi1, which were responsible for the molecular suppression of RORγt and STAT3, were up-regulated by rapamycin. These results point toward a pivotal view to treatment of ALI through weakening the proliferation of Th17 cells with rapamycin.

Acute lung injury (ALI), which was first described in 1967, is now defined as mild acute respiratory distress syndrome (ARDS), with a P/F ration of <300 mmHg (40 kPa) and PEEP/CPAP levels of ≥5 cmH_2_O, according to the Berlin Definition^1^. Proteinaceous pulmonary edema and severe hypoxemia are outstanding pathogenic characteristic of ARDS, and they are at the root of neutrophil-induced inflammation^2^. However, the half-life of neutrophils is approximately 6~8 hours *in vivo*^3^. Accumulated evidence indicates that neutrophil recruitment occurs later, after tissue injury, and that it requires prolonged high concentrations of chemokines. The immune response activation is thought to occur earlier.

T helper 17 (Th17) cells, a recently detected effective subset of CD4^+^T cells and the major source of IL-17 (IL-17A) and IL-22, play an important role in defending a host against microorganisms, such as staphylococcus^4^,^5^. Patients with infection-induced ALI/ARDS appeared to have obvious activation and proliferation of T-cells, particularly the presence of Th17 cells^6^. Moreover, IL-17 potently induces the production of IL-8 and G-CSF from epithelial cells and fibroblasts, thereby leading to neutrophil activation and recruitment. Meanwhile, IL-17R knockout (KO) mice demonstrated greater dissemination of K. pneumonia and higher mortality compared with control mice^7^. Similar to the IL-17RA^−^/^−^ mice, the IL-17A^−^/^−^ mice had significantly lower levels of G-CSF and CXCL1 in their bronchoalveolar lavage fluid (BALF) at 24 h compared to the WT mice^8^. Interestingly, the Th17-mediated immune response is reported not fully activated in ARDS patients^9^.

The differentiation of Th17 cells is moderated by many pathways. STAT3 and RORγt are two of the most important nuclear transcription factors in this regulation process^10^,^11^. Coincidentally, both nuclear transcription factors are up-regulated through activation of the mammalian target of rapamycin complexes 1 (mTORC1)^12^,^13^. Many signs indicated that the mTORC1 molecule plays a key role in the polarizing progress of Th17 cells.

The mTORC1 pathway plays a critical role in cell growth, proliferation, and/or survival. As an efficient inhibitor of mTORC1, rapamycin plays a role as an anticancer agent in clinical practice. Researchers also hope that rapamycin can mediate inflammatory disease by regulating effector cells. In fact, the effect of suppressing mTORC1 during ALI has been controversial. Some researchers have reported that mTORC1 inhibited by endogenous inhibitor Rtp801 could exacerbate lung inflammation^14^,^15^. Jill and colleagues have also found that rapamycin augmented LPS-induced lung injury through a mechanism involved in STAT1-related apoptosis^16^. However, some studies have demonstrated that rapamycin protects ALI mice through different mechanisms, such as by blocking the NF-κB pathway, enhancing autophagy, promoting the expansion of Treg cells or inhibiting the production of inflammatory mediators^13^,^17^,^18^,^19^.

What is the role of Th17 cells in neutrophil-mediated diseases, such as ALI/ARDS? Is it regulated by rapamycin through the mTORC1 pathway? What is the effect if LPS-induced ALI mice treated with rapamycin? These questions stimulated this research.

## Results

### IL-17 and IL-22 levels were elevated in ARDS patients, and high levels were maintained in nonsurvivors after 7 days of treatment

**Figure 1:**
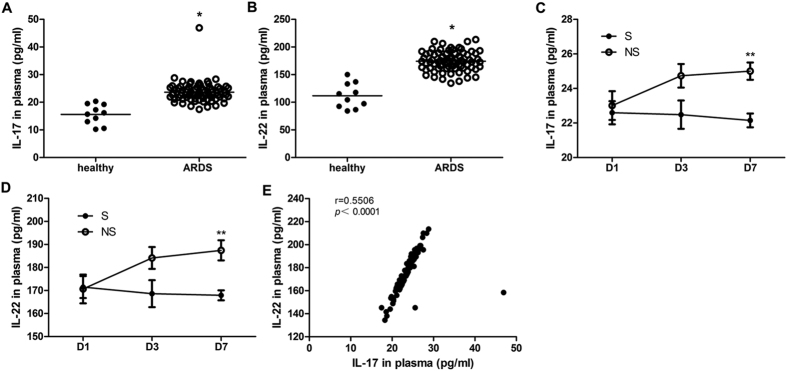
The plasma levels of Th17 linear cytokines in patients with sepsis-related ARDS. (**A**,**B**) The plasma levels of IL-17 and IL-22 in ARDS patients and healthy subjects based on D1 data (*p < 0.05, compared with healthy subjects). The lines indicate the mean value. (**C**,**D**) The plasma levels of IL-17 and IL-22 measured at different time points for survivors and nonsurvivors (**p < 0.01, compared with survivors). The error bars indicate SEM. (**E**) Correlation of two cytokines calculated with linear regression. The *r* represents Pearson’s correlation coefficients, and a *p* value of less than 0.05 was considered to be statistically significant.

IL-17 and IL-22 are Th17 linear cytokines. To further confirm the pro-inflammatory role of Th17 cells, we measured the plasma levels of these two cytokines in 79 patients with sepsis-induced ARDS. Moreover, we also compared the cytokine levels of 9 nonsurvivors and 10 survivors at three different points. As expected, the IL-17 and IL-22 levels in the ARDS patients increased significantly (Fig. 1A,B). The highly positive correlation between IL-17 and IL-22 also indicates that in most cases, the two cytokines stemmed from the same source (Fig. 1E). Compared with the survivors, the nonsurvivors retained high concentrations of IL-17 and IL-22 until Day7 (D7) (Fig. 1C,D). Uncontrolled inflammation may be a risk factor for poor prognosis in patients with ARDS. Managing the proliferation and differentiation of Th17 cells is thought to be an effective treatment. The baseline character and clinical data are shown in the supplement data (S.Tables 2 and 3).

### Inflammation characteristics in an ALI murine model: neutrophils increased later than the production of inflammatory mediators

**Figure 2:**
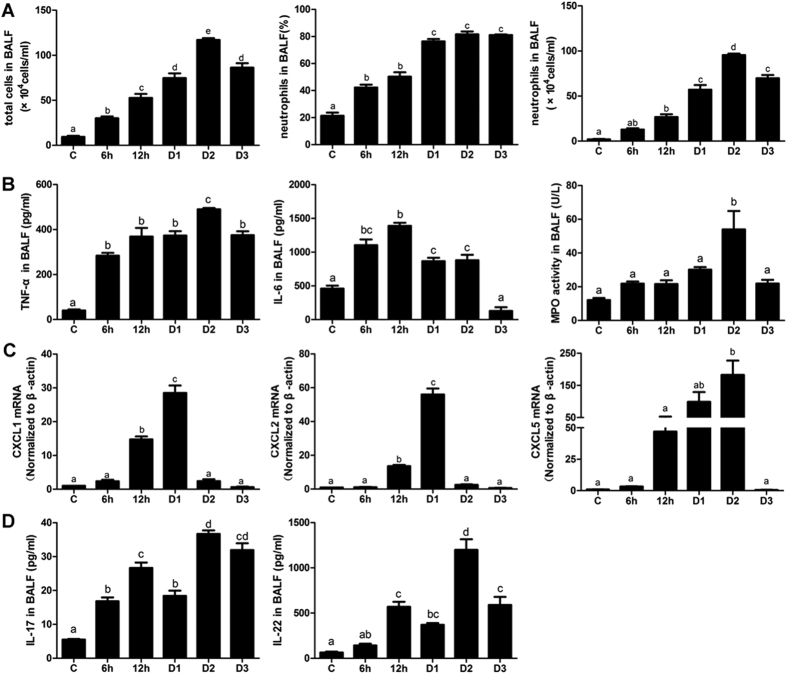
Inflammatory characteristics of the ALI murine model. (**A**) Total inflammatory cells and neutrophils in BALF. (**B**) Inflammatory mediators, including TNF-α, IL-6 and MPO activity in BALF. (**C**) The mRNA expression of chemokines, including CXCL1, CXCL2 and CXCL5. (**D**) Th17-linear cytokines, including IL-17 and IL-22 in BALF. The error bars indicate SEM. The different superscript letters (a–e) represent a significant difference in these columns (*p* < 0.05). All mice were wild type, with 4~6 mice per group.

The total cell numbers in BALF started to increase 6 hours post-LPS and peaked two days later. Approximately 80% of the neutrophils were maintained from Day1 (D1) to Day3 (D3). The absolute value of neutrophils was at the highest level on Day2 (D2) (96 × 10^4^ cells/ml, Fig. 2A). The inflammatory mediators significantly increased from the 6 h time point (or earlier). The IL-6 level continued to be high until D2, although it primarily increased during the early phase. Meanwhile, TNF-α did not demonstrate any particular difference in terms of time. MPO reflects neutrophil activity, and it peaked at D2, similar to the neutrophils (Fig. 2B). Continued neutrophil recruitment requires an external driving force; therefore, we focused on the expression of chemokines. CXCs began to increase beginning at 12 hours post-LPS, primarily in the lung tissue, and then peaked at different time points. Interestingly, only CXCL5 maintained a high level from 12 h to D2 (Fig. 2C). CXCL5 may be the most important chemokine involved in recruiting neutrophils to the target area. Note that the IL-17 and IL-22 levels were obviously decreased at D1 before peaked at D2 (Fig. 2D).

### Th17 cells evaluated in an LPS-induced ALI murine model

**Figure 3:**
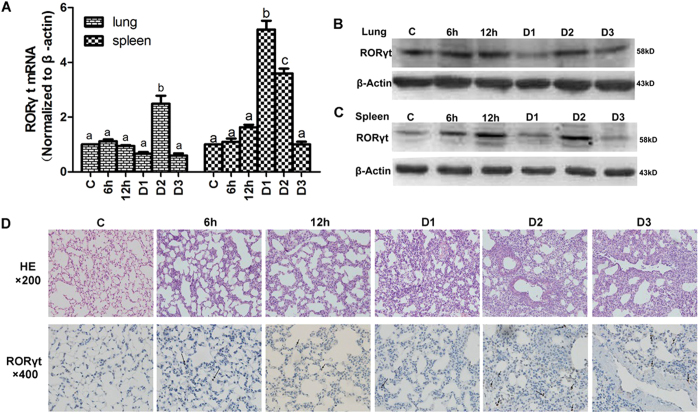
Th17 cells are highly expressed at D2 during ALI development. (**A**) The expression of RORγt mRNA in lung and spleen tissue at different time points. The error bars indicate SEM. The different superscript letters (a–c) represent a significant difference of these columns (*p* < 0.05). (**B**,**C**) Western blot analysis tested the expression of RORγt protein in the lungs and spleen, respectively. (**D**) Histopathological and immunohistochemistry findings (HE: ×200; IHC: ×400). The tan color indicates RORγt-positive staining. Arrows indicate positive cells. All mice were wild type, with 4~6 mice per group.

Hypothesizing that Th17 cells are the main source of IL-17 and IL-22, we explored the expression of RORγt, the specific nuclear transcription factor of Th17 cells, in an LPS-induced ALI murine model. As the primary peripheral immune organ, the spleen is where effector T cells activate and proliferate. In the present study, the RORγt gene was amplified earlier in the spleen, increasing by approximately 5 times on D1 (Fig. 3A). The lungs are the target organs where ALI occurred. However, the proliferation of Th17 cells occurs later in the lungs than the spleen, and the RORγt gene reached peak amplification (2.6 times) at D2 (Fig. 3A). Slightly inconsistent with the qPCR results, the highest level of inducible RORγt protein in the spleen and lungs appeared at D2 point (Fig. 3B,C). Compared with the control group, LPS triggered inflammatory cell infiltration and destroyed the normal structure of the lung tissue. The tissue damage demonstrated higher severity and wider lesions at D2 (Fig. 3D). The immunohistochemical results also demonstrated that RORγt positive cells were much higher at D2 during ALI development (Fig. 3D).

### Complete lack of IL-17 failed to accumulate and activate neutrophils

**Figure 4:**
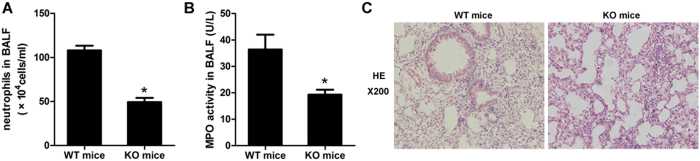
Neutrophils mediated inflammation attenuated in IL-17^−^/^−^ mice. (**A**) Neutrophils in BALF between the WT and KO mice. (**B**) MPO activity in BALF between the WT and KO mice. (**C**) Infiltrated neutrophils and the degree of inflammation were obviously attenuated in the lung tissue of the mice completely lacking IL-17. (**A**,**B**) *p < 0.05, compared with the WT mice. The error bars indicate SEM. All mice were harvested on D2, and there were 3~6 mice per group.

We used IL-17 KO mice to evaluate the role of IL-17 in neutrophil recruitment. The data showed that the number of neutrophils decreased in the KO mice (Fig. 4A). Meanwhile, the MPO activity also decreased (Fig. 4B). As a result, the neutrophil-mediated inflammation was obviously attenuated in the IL-17 deficiency mice (Fig. 4C). The complete lack of IL-17 partially inhibited the recruitment and activation of neutrophils and protected the lung tissue from injury.

### IL-22 showed a stronger inflammatory promoting effect

**Figure 5:**
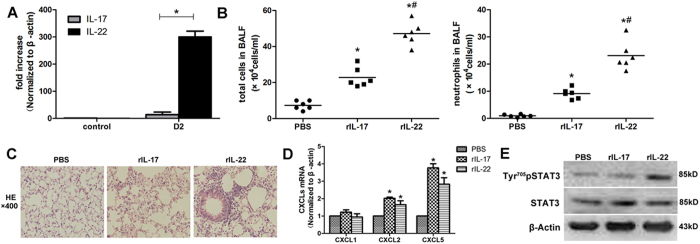
Recombinant IL-22 induced more severe pulmonary inflammation than recombinant IL-17. (**A**) The expression of IL-17 and IL-22 mRNA in LPS-challenged mice at D2. *p < 0.05, compared with the expression of IL-17. (**B**) Total inflammatory cells and neutrophils in the BALF of the mice treated with recombinant cytokines. *p < 0.05, compared with the PBS-treated group, ^**#**^*p* < 0.05, compared with the rIL-17-treated group. (**C**) The histopathological findings demonstrated severe neutrophils infiltrated in rIL-22 treated mice (HE: ×400). (**D**) CXCLs amplification in qPCR in WT mice treated with recombinant cytokines. *p < 0.05, compared with the PBS-treated group. (**E**) Recombinant IL-22 increased the phosphorylation level of STAT3. Mice treated with recombinant cytokines (**B**–**E**) were harvested on D1. The error bars indicate SEM. All mice were wild type, with 3~6 mice per group.

IL-17 and IL-22 are homologous factors that are mainly secreted by Th17 cells. The mRNA amplification of IL-22 was more striking than that of IL-17, according to qPCR (Fig. 5A). Previous studies have reported that IL-22 played conflicting roles in the inflammatory response. To further explore the role of IL-22 in lung injury, 1 μg recombinant mouse IL-17and IL-22 was given intratracheal. IL-22 triggered many more inflammatory cells in BALF, particularly neutrophils (Fig. 5B). The histopathological findings also demonstrated that redundant inflammatory cells aggregated in the lung tissue (Fig. 5C). However, the expression levels of CXCL1, CXCL2 and CXCL5 induced by rIL-22 were not significantly different from those induced by rIL-17 (Fig. 5D).The reason for this discrepancy might be that rIL-22 plays a role in the activation of STAT3. The level of phosphorylated STAT3 was significantly increased in the lung tissue of the mice treated with rIL-22 (Fig. 5E).

### Rapamycin attenuated lung inflammation through down-regulation of phosphorylated mTORC1

**Figure 6:**
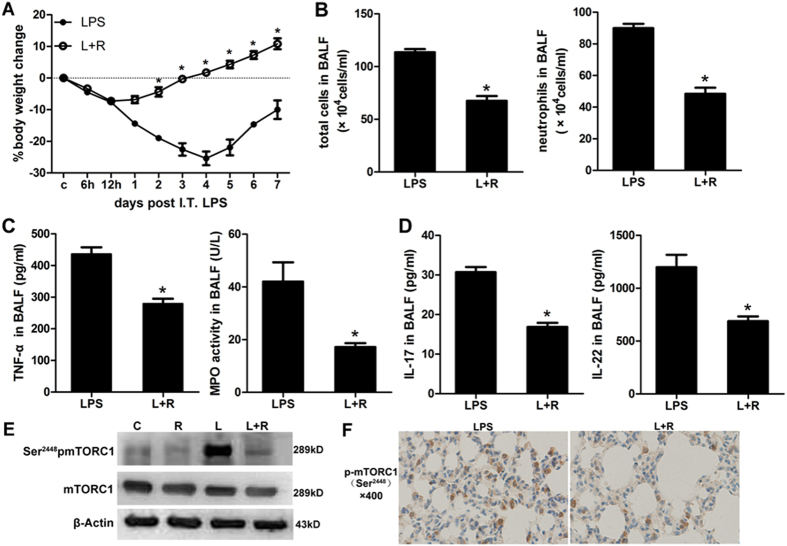
Rapamycin attenuated lung inflammation through down-regulation of phosphorylated mTORC1. (**A**) Body weight changes in the LPS challenged mice treated with or without rapamycin. (**B**) Inflammatory cells in the BALF of mice treated with or without rapamycin. (**C**) Inflammatory mediators in the BALF of mice treated with or without rapamycin. (**D**) Th17-linear cytokines including IL-17 and IL-22 in the BALF of mice treated with or without rapamycin. (**E**) Weston blot analysis tested the protein level of phosphorylated mTORC1. (**F**) Immunohistochemistry analysis tested the protein level of phosphorylated mTORC1. The tan color indicates Ser^2448^pmTORC1-positive staining. R: rapamycin; L: LPS; C: control; I.T.: intratracheal. The mice were harvested on D2. *p < 0.05, compared with LPS treated group. Error bars indicate SEM. All mice were wild type, with 3~5 mice per group.

Our previous study found that rapamycin exerts a protective effect on ALI mice. The current study further confirmed that mice treated with rapamycin lose less weight and recover from ALI faster (Fig. 6A). The proportion of neutrophils decreased by approximately 50% (Fig. 6B). Moreover, the inflammatory mediators in BALF, including TNF-α, MPO activity, IL-17 and IL-22, were markedly reduced (Fig. 6C,D). In the rapamycin-treated mice, the inflammation relief was accompanied by a lower expression level of phosphorylated mTORC1 (Fig. 6E,F).

### Inhibition of the PI3K/AKT-mTORC1 axis downregulated the expression of Th17 cells

**Figure 7:**
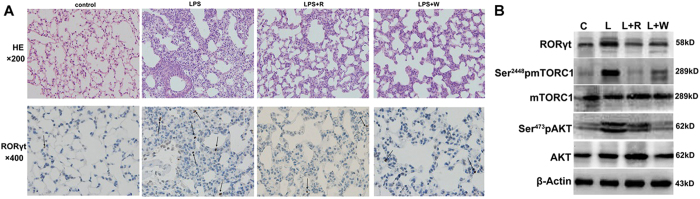
Inhibition of mTORC1 or its upstream all downregulated the expansion of Th17 cells. (**A**) Histopathological and immunohistochemistry findings (HE: ×200; IHC: ×400). The tan color indicates RORγt-positive staining. The arrows indicate positive cells. (**B**) The protein levels of PI3K/AKT- mTORC1 pathway and RORγt in injured lungs. (**C**) control; L: LPS; R: rapamycin; W: wortmannin. The mice were harvested on D2. All mice were wild type, with 3~5 mice per group.

PI3K/AKT is located in the upper reaches of mTORC1, and it could be effectively inhibited by wortmannin. The number of RORγt positive cell in the LPS group was the highest, and the inhibition of either PI3K/AKT or mTORC1 could significantly decrease this number (Fig. 7A). The expression levels of RORγt and phosphorylated mTORC1 were unchanged in the presence of wortmannin compared to rapamycin (Fig. 7B). These results indicated Th17 differentiation in the lungs regulated by the PI3K/AKT-mTORC1 axis during ALI.

### Rapamycin impaired Th17 differentiation but not through up-regulating Treg cells

**Figure 8:**
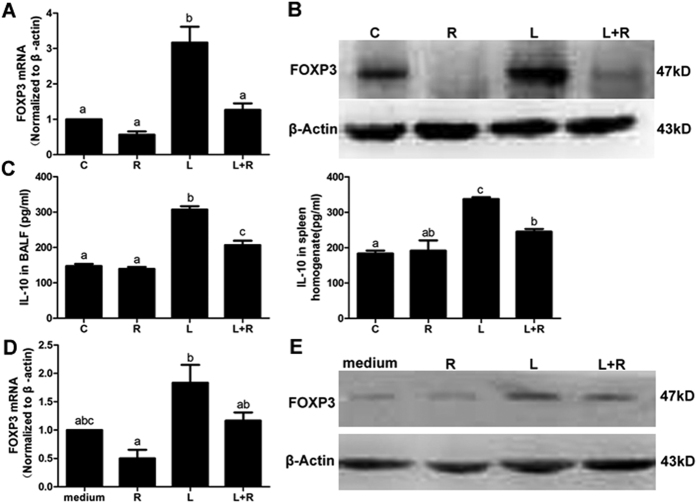
Rapamycin did not promote Treg cells proliferation in *vivo* and *vitro*. (**A**) The expression of FOXP3 mRNA in the lungs. (**B**) The protein level of FOXP3 in the lungs. (**C**) The level of IL-10 in the BALF and spleen homogenate. (**D**) The expression of FOXP3 mRNA in mixed lymphocytes reaction. (**E**) The expression of FOXP3 protein in mixed lymphocytes reaction. C: control; L: LPS; R: rapamycin. The mice (**A**–**C**) were harvested on D2. The different superscript letters (a–c) represent a significant difference in these columns (P < 0.05). All mice were wild type, with 3~5 mice per group.

Treg cells maintain the immune homeostasis by inhibiting the overreaction of effector T cells. Studies have reported that rapamycin promotes the differentiation of Treg cells, while weakening the expression of Th17 cells^20^,^21^,^22^. The current results are not consistent with these reports because we did not effectively amplify Treg cells with rapamycin *in vivo* and *in vitro*. The FOXP3 level in the mice treated with rapamycin was significantly decreased compared with the model group (Fig. 8A). Moreover, as an anti-inflammatory mediator secreted by Treg cell, IL-10 was remarkably reduced in BALF and spleen homogenates (Fig. 8B). In the mixed lymphocyte reaction, rapamycin- and LPS-treated DCs unsuccessfully expanded the Treg cells. Both the mRNA and protein levels of FOXP3 were not different from the control group (Fig. 8C).

### Rapamycin inhibited the proliferation of Th17 cells through upregulation of the expression of SOCS3 and Gfi1

**Figure 9:**
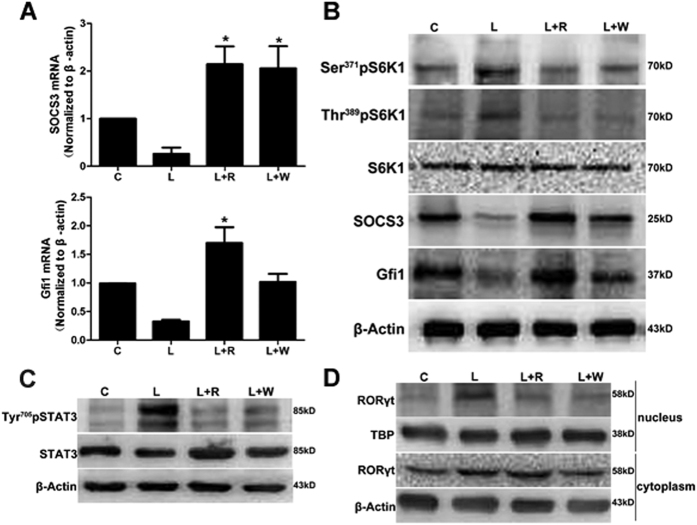
Rapamycin inhibited the proliferation of Th17 cells by up-regulating the expression of SOCS3 and Gfi1. (**A**) The expression of SOCS3 and Gfi1 mRNA in the lungs. *p < 0.05, compared with the LPS-treated group. The error bars indicate SEM. (**B**) The protein levels of S6K1, SOCS3 and Gfi1 in the lungs. (**C**) The protein level of phosphorylated and total STAT3. (**D**) Differential expression of RORγt in cytoplasm and nucleus. C: control; L: LPS; R: rapamycin; W: wortmannin. The mice were harvested on D2. All mice were wild type, with 3~5 mice per group.

As an important transcription factor that promotes proliferation, STAT3 is a positive regulatory element for Th17 polarization. A recent study has shown that SOCS3 could block the phosphorylation of STAT3. Either the inhibition of mTORC1 or its upstream could trigger the same effect of decreased STAT3 phosphorylation through upregulation of SOCS3 (Fig. 9A–C).

Gfi1, as a novel negative regulator of Th17 differentiation, blockaded the nuclear localized site of RORγt without affecting its expression^23^. Furthermore, the expression of Gfi1 is regulated downstream of the mTORC1-S6K1 axis^12^. In this study, the expression of Gfi1 at D2 was increased in the presence of rapamycin or wortmannin at the mRNA or protein level (Fig. 9A). As expected, the phosphorylation level at Ser371 and Thr389 of S6K1 were both down-regulated by rapamycin and wortmannin. Meanwhile, the protein expression of Gfi1 was obviously increased (Fig. 9B). As a result, RORγt located in the nucleus was significantly reduced in the mice treated with rapamycin and wortmannin (Fig. 9C).

## Discussion

ALI is defined as an inflammatory disease. Neutrophils and inflammatory mediators were markedly increased during the early phase. Recent studies have suggested that the immune regulation disorder may be an important factor in initiating inflammation. Treg and Th17 cells, which belong to CD4^+^ T cells, have gained much attention. Treg, characterized as a CD4^+^CD25^+^FOXP3^+^ T cell, is the master of immune system through a) suppressing a wide array of effector immune cells, including Th cells, B cells dendritic cells and macrophages, and b) secreting of immunosuppressive cytokines, such as TGF-β and IL-10^24^,^25^. Interestingly, Treg cells have been activated during ALI development^26^. A high proportion of Treg cells even became a risk factor for predicting poor outcome of ARDS patients^27^. In our study, the increased expression of IL-10 and FOXP3 all demonstrated the proliferation of Treg. However, rapamycin attenuated the lung injury induced by LPS along, with down-regulated FOXP3 levels, which suggests that rapamycin plays a treatment role through another T cell, Th17 cell.

Although Th17 cells have been demonstrated to be important in infectious diseases, their role in ALI/ARDS is controversial. A two microarray dataset analysis of ARDS trials with GeneChip showed that the Th17 immune response might not be successfully triggered according to down-regulated key Th17 transcription factors, STAT3 and RORα^9^. Another research reveals that ALI/ARDS due to infection is associated with a high level of Th17-cell activation and proliferation^6^. In the present murine model of ALI, we found that Th17 cells were over expressed. However, the proliferation was biphasic instead of a linear change. We speculate that early activation of Th17 cells belongs to the natural type. However, the later activation of Th17 cells is labeled as the inducible type due to the proliferation and polarization of naïve CD4^+^ T cells from the immune organs.

During the differentiation of Th17 cells, IL-6 was thought to be a key component, combined with TGF-β^28^. Consistent with other reports, its sphere of influence started from the initial of injury until 2 days after the LPS challenge^26^. We concluded that IL-6, at minimum, plays two roles in the development of ALI. However, the emphasis differs during different periods. Note that IL-6 recruits neutrophils directly in the early stage. In the later stages, IL-6 performed an immune regulator function, acting as a transcriptional activator^29^. Of course, there is no clear boundary between the two periods. In the presence of IL-6, the expression of FOXP3 was abrogated while RORγt initiated, thereby promoting the differentiation of Th17 cells^30^,^31^.

In our study, the percentage of neutrophils started to increase at the 6 h time point and reached and maintained approximately 80% at D2 and D3. Consistent with the growth trend of neutrophils in BALF, inflammatory mediators, such as TNF-α and IL-17, peaked on D2. Previous studies have shown that IL-17 alone or with TNF-α can successfully increase neutrophils by inducing chemokines CXCL1/2/5/8^32^,^33^. IL-17RA KO mice that lacked the response to several IL-17 cytokines showed decreased neutrophils due to reduced CXCL1/2^34^. We examined the expression of CXCL1/2/5. Our results supported the results of Liu and colleagues; early rising and sustained high expression levels suggest that CXCL5 is more important than CXCL1/2^35^. Both recombinant IL-17 and IL-22 induced CXCL2/5 expression, and the CXCL5 level increased more obviously. Hypothesizing that IL-17 and IL-22 are primarily secreted by Th17 cells, we speculated that Th17 cells facilitate neutrophil recruitment and migration^36^.

We also demonstrated that IL-22 showed stronger pro-inflammatory abilities by recruiting neutrophils that infiltrated to the lungs. STAT3 activation is the logical explanation for this phenomenon. Phosphorylated STAT3, the activated form of STAT3 protein, provides a rapid membrane for the nucleus mechanism regulating the expression of nuclear translation factor. STAT3 also has the ability to promote the expansion of Th17 cells^37^,^38^. IL-22 is the key component of the positive feedback loop formed by STAT3 activation and Th17 cell proliferation. Breaking the pro-inflammatory circular chain may provide effective treatments in the future.

STAT3 expression was also regulated by the mTORC1 pathway. The mTORC1 molecule plays a critical role in generating a coordinated response to the cellular environment (cell growth, proliferation, and/or survival) through modulation of protein synthesis. After the LPS challenge, mTORC1 was significantly activated in lungs along with the overexpressed nuclear translation factors of Th17 cells. MTORC1 is likely to be an important factor of Th17 cell proliferation. Our experiment found that Th17 cells were significantly decreased either through inhibition of mTORC1 (by rapamycin) or its upstream (PI3K/AKT, by wortmannin).

To evaluate the possible mechanism through which rapamycin protects lung injury, we first excluded the possibility that it regulated Th17 differentiation through the expansion of Treg cells. The exact effect of rapamycin in our experiment was to increase the molecular suppression level, including SOCS3 and Gfi1. As expected, the high expression of SOCS3 can inhibit the phosphorylation level of STAT3. Previous studies have reported that Gfi1 is the downstream of the mTORC1-S6K1 axis, which can regulate the nuclear localization of RORγt. In mice treated with rapamycin, there was a down-regulation of the RORγt total protein, as well as blocked transcription from the cytoplasm to nuclear.

HIF-1α, downstream of the mTORC1 molecule, revealed another regulatory method. Following activation of mTORC1, overexpressed HIF-1α enhances Th17-related gene including STAT3 and RORγt expression^39^,^40^. Moreover, it could promote Th17 proliferation by increasing glycolytic activity, which is required for rapid T cell expansion^41^. In addition, there is a positive-feedback loop between mTORC1 and HIF-1α during Th17 differentiation, which is induced by hypoxia, independently of PI3K/AKT^42^.

MTORC1 is the hub that links several distinct mechanisms regulating Th17 differentiation. MTORC1 dysfunction through the deletion of Rheb or Raptor impairs Th17 differentiation^12^,^43^. We believed that focusing on mTORC1 to regulate Th17 cell-mediated inflammation could be a new and useful treatment target.

Our work provides new insights to treat ALI/ARDS through immune system regulation. Although our experiments have demonstrated that rapamycin can protect the ALI murine model by reducing the differentiation of Th17 cells, there are still some questions that must be answered. What causes the decrease in Th17 and Treg cells after rapamycin treatment? Is the whole immune system inhibited? What about the status of rapamycin, which slowed the life process by inhibiting protein synthesis in this experiment? We will conduct a thorough study of these issues.

## Materials and Methods

### Animals and experimental protocol

Male C57BL/6 and BALB/c mice, 6–8 wks of age and free of murine-specific pathogens, were obtained from the Department of Laboratory Animal Center of Chongqing Medical University. IL-17 knockout (IL-17 KO or IL-17^−^/^−^) mice with a C57BL/6 background were kindly provided by Yibing Yin (Key Laboratory of Diagnostic Medicine, Chongqing Medical University, China) and the Medical School of Tokyo University (Tokyo, Japan). The mice were housed throughout the experiments in a laminar flow cabinet, and all experimental protocols involving animals were approved by the Ethics Committee of the Second Affiliated Hospital of Chongqing Medical University and implemented in accordance with the instructions of the National Institutes of Health Guide for the Care and Use of Laboratory Animals.

Before the experiments started, the mice were following a minimum facility acclimatization period of 7 days. The mice were anesthetized through intraperitoneal administration of 4% chloral hydrate (0.1 ml/10 g) and intratracheally instilled with 3 mg/kg LPS (Sigma, prepared from Escherichia coli 0111:B4) in 50 μL PBS or sterile PBS alone (as the control group). Rapamycin (5 mg/kg) was intraperitoneally administered in a total volume of 200 μL for 3 consecutive days before LPS was administered^44^. To investigate the role of PI3K/AKT pathway, wortmannin, the inhibitor of catalytic subunit p110 was injected, as previously described^45^. Briefly, 16 μg/kg wortmannin was intravenously administered 30 minutes prior to the LPS administration. To verify the role of IL-22 and IL-17, we gave healthy mice 1μg mouse recombinant IL-22, IL-17 or PBS as a control^46^. The experiment protocol is shown in the supplemental data (S. Figure 1).

### Patients

The cohort consisted of 79 sepsis-related-ARDS patients admitted to the respiratory intensive care unit (RICU) between May 2012 and May 2015 at the Second Affiliated Hospital of Chongqing Medical University and the Yongchuan Affiliated Hospital of Chongqing Medical University. The inclusion criteria, primarily as previously described^47^, were performed according to the Berlin definition^1^. The following exclusion criteria were applied: 1) 18 years of age and younger, 2) previously underwent immunosuppressant therapy, 3) received long term glucocorticoid therapy (0.5 mg/Kg/D, ≥1 months), 4) confirmed or suspected malignant tumor history, and 5) previously included in other studies. Blood samples were collected within 24 hours and at another 2 time points after the patient met the inclusion criteria. Briefly, peripheral venous blood samples were drawn on an agreed upon time point and centrifuged at 3000 rpm for 15 minutes. The plasma was stored at −80 °C for ELISA testing. The plasma levels of IL-17 (Shanghai Yanhui Biological Technology Co., Ltd) and IL-22 (Shanghai Yanhui Biological Technology Co., Ltd) were measured using commercially available sandwich ELISA kits.

The study protocol was reviewed and approved by the local Institutional Review Board, and written informed consent was obtained from either the patient or from each patient’s next of kin or legal representative before study enrollment.

### Mixed lymphocytes reaction

DCs were generated and cultured according to the previously procedure described procedure^48^. Briefly, bone marrow cells were obtained from the femurs and iliac bones of BALB/c mice and cultured with completive medium (DC-CM; RPMI 1640 containing 10% heat-inactivated fetal calf serum [FCS], 50 mM 2-ME, 2 mM L-glutamine, 100U/ml penicillin, 100 mg/ml streptomycin) (GIBCO), 10 ng/ml recombinant mouse GM-CSF, and 10 ng/ml recombinant mouse IL-4 (R&D Systems). On Day 6, DCs were purified using CD11c+ microbeads (Miltenyi Biotec), pulsed with RPM or LPS for 24 hours and washed three times with PBS. As a control, DCs were cultured only with medium. CD4^+^ naïve T cells were generated from the spleen cells of C57BL/6 mice and purified using microbeads from Miltenyi Biotec. CD4^+^ naïve T cells and 7-day-long DCs were co-cultured with ConA(Sigma) for 6 hours. Then, cells were collected for WB and qPCR testing.

### Evaluation of lung histology

The lungs were fixed in 10% formalin for 48 hours and embedded in paraffin, as previously described^49^. Longitudinal sections (4-mm thick) were cut from the left lobe, placed on glass slides, deparaffinized, and sequentially stained with H&E (Richard-Allan Scientific).

### Immunohistochemistry

As previously described, deparaffinized and PBS-washed sections were incubated with 3% H_2_O_2_ in PBS (pH7.6) for 15 min to block endogenous peroxides. Background non-specific binding was reduced by incubating with 1% BSA in PBS for 60 min at room temperature. The sections were incubated with primary antibody against RORγt (Abcam, 1:300) or p-mTORC1 (Abcam, 1:200) overnight at 4 °C. After washing, the sections were incubated with biotinylated IgG (Abcam) for 15 min at 37 °C and reacted with avidin–biotin–peroxidase complex (Sigma) for15 min. Subsequently, the sections were stained with DAB (ZSGB-BIO, China) for 5 min, followed by hematoxylin staining for 2 min.

### Evaluation of lung inflammation

BALF was performed to determine the total cell numbers using a hemocytometer. Smears of BALF cells were stained with Giemsa to determine the neutrophil counts. The concentrations of IL-17 (R&D Systems,USA), IL-22 (R&D Systems,USA),TNF-α (R&D Systems,USA), IL-6 (Neobioscience Technology Co., Ltd, China) and MPO activity (Colorful Gene, China)in the BALF were measured with enzyme immunoassays, according to the manufacturer’s protocols.

### Quantitative real time-PCR

Total RNA was isolated from the right lung tissues, according to the manufacturer’s protocol (TaKaLa, Japan). The relative mRNA levels of each sample were determined using NanoDrop2000 (Thermo, USA). Quantitative RT-PCR (qPCR) analysis was performed using the SYBR Green (TaKaLa, Japan). The relative gene expression was calculated using CFX manager software (Bio-Rad, USA), using the comparative Ct (ΔΔCt) method, with β-Actin as a reference gene. The primer sequences are shown in the supplemental data (S. Table1).

### Western blot analysis

The protein levels of relative genes were analyzed with Western blot. Total protein and phosphorylated protein were extracted according to the manufacturer’s protocol (KeyGEN Bio TECH Co., Nanjing, China). Briefly, 100 mg lung tissues were lysed and homogenized with 1000 μL RIPA buffer supplemented with phosphatase inhibitors and/or protease. After being mixed and incubated on ice for 15 minutes, the homogenate was centrifuged at 12000 × g for 15 min at 4 °C. A BCA kit used to determine the concentration of supernatant. An equal amount of protein (100 μg) was separated through electrophoresis on SDS-PAGE and then transferred to polyvinylidene fluoride membranes. After blocking with 5% non-fat milk or BSA for 1 h, the membrane was incubated with antibodies overnight at 4 °C. The membrane was washed 3 times with PBST, and then the second antibody was incubated (Abcam, 1:5000) at 37 °C for 1 h. Protein bands were exposed with enhanced ECL kits and analyzed with Quantity One software (Bio-Rad, Hercules, CA, USA).

For Western blot analysis, the following primary Abs were used: anti-RORγt (Abcam, 1:500), anti-FOXP3 (Abcam, 1:1000), anti-pmTORC1 Ser2448 (Abcam, 1:1000), anti-pS6K1 Ser371 and Thr389 (Cell Signaling Technology, 1:500), anti-S6K1 (Cell Signaling Technology, 1:500), anti-pSTAT3 Tyr705 (Cell Signaling Technology, 1:500), anti-pAKT Ser473 (Cell Signaling Technology, 1:4000), anti-SOCS3 (BioWord, 1:1000), anti-Gif1 (BioWord, 1:1000) and β-Actin (Abcam, 1:5000).

### Statistical analysis

GraphPad 5.0 prism software was used to perform all statistical analyses and to draw the graphics. Data were analyzed through one-way analysis of variance with the Bonferroni multiple comparison test. An unpaired Student’s *t*-test was used for the comparison between two groups. Data were shown as the mean ± S.D. A value of *p* < 0.05 was considered to be statistically significant.

## Additional Information

**How to cite this article**: Yan, Z. *et al.* Rapamycin attenuates acute lung injury induced by LPS through inhibition of Th17 cell proliferation in mice. *Sci. Rep.* **6**, 20156; doi: 10.1038/srep20156 (2016).

## Supplementary Material

Supplementary Information
