# Supplementary material for: Rapamycin attenuates acute lung injury induced by LPS through inhibition of Th17 cell proliferation in mice
Source: Sci Rep. 2016 Feb 18;6:20156. doi: 10.1038/srep20156 (PMC4757870; doi:10.1038/srep20156)
Supplement: Supplementary Information [file srep20156-s1.doc]

**Supplementary Information**

**Rapamycin attenuates acute lung injury induced by LPS through inhibition of Th17 cell proliferation in mice**

Zhao Yan, Zhang Xiaoyu, Song Zhixin, Qi Di, Deng Xinyu, Xia Jing, He Jing, Deng Wang, Zhong Xi, Zhang Chunrong & Wang Daoxin

**Figure 1**

-D3 -D2 -D1 -30min 0

6h

12h

D1 D2 D3

I.T. LPS, rIL-17 or rIL-22

wortmannin

rapamycin

**Figure 1. Experimental protocol.** The mice were anesthetized and intratracheally instilled with 3 mg/kg LPS, 1μg mouse recombinant IL-22 or IL-17 in 50 μL PBS at D0. Wortmannin (16μg/kg) was administered intravenously 30 minutes prior to the LPS administration. Rapamycin (5 mg/kg) was administered intraperitoneally for 3 consecutive days in a total volume of 200μL before LPS was administered. The mice were harvested at different time points for further detection.

**Table 1.** Relevant gene names and sequences

|  | Sense(5' →3') | Antisense(5'→3') |
| --- | --- | --- |
| β-actin | CCTGAGGCTCTTTTCCAGCC | TAGAGGTCTTTACGGATGTCAACGT |
| RORγt | TCCACTACGGGGTTATCACCT | AGTAGGCCACATTACACTGCT |
| FOXP3 | CACCTATGCCACCCTTATCCG | CATGCGAGTAAACCAATGGTAGA |
| CXCL1 | CTGGGATTCACCTCAAGAACATC | CAGGGTCAAGGCAAGCCTC |
| CXCL2 | CCAACCACCAGGCTACAGG | GCGTCACACTCAAGCTCTG |
| CXCL5 | GTTCCATCTCGCCATTCATGC | GCGGCTATGACTGAGGAAGG |
| IL-17 | TCAGCGTGTCCAAACACTGAG | CGCCAAGGGAGTTAAAGACTT |
| IL-22 | ATGAGTTTTTCCCTTATGGGGAC | GCTGGAAGTTGGACACCTCAA |
| SOCS3 | TGCGCCTCAAGACCTTCAG | GCTCCAGTAGAATCCGCTCTC |
| Gfi1 | AGAAGGCGCACAGCTATCAC | GGCTCCATTTTCGACTCGC |

**Table 2. Baseline characteristics of 79 patients with sepsis-related ARDS**

| Characteristics | ARDS (n =79) |
| --- | --- |
| Age, year | 67(54-73) |
| Male sex, n (%) | 58(73.4) |
| APACHE II score | 21(17-26) |
| SAPS II score | 43(33-51) |
| PaO_2_/FiO_2_ratio | 162(120-193) |
| Hospital stay, day | 16(9-26) |
| ICU stay, day | 8(3-15) |
| WBC count,×10^3^/mm^3^ | 13.28(8.16-19.48) |
| PCT(pg/ml) | 2.53(0.48-6.57) |
| Predisposing conditions, n (%) |  |
| Bacteremia | 28(35.4) |
| Pneumonia | 51(64.6) |
| Comorbidity, n (%) |  |
| Obstructive airway disease | 24(30.4) |
| Cardiovascular disease | 23(29.1) |
| Diabetes | 21(26.6) |
| Hypertension | 17(21.5) |
| Cancer history | 6(7.6) |
| MODS, n (%) |  |
| Dysfunction ≥3 organs | 22(27.8) |
| Septic shock | 35(44.3) |
| Renal failure | 11(13.9) |
| Hepatic failure | 9(11.4) |

Data are presented as median (IQR) or No. (%).

ARDS = acute respiratory distress syndrome; APACHE = acute physiology and chronic health evaluation; SAPS II= new simplified acute physiology score; MODS = multiple-organ dysfunction syndrome; WBC = white blood cell; PCT= procalcitonin.

**Table3.** Characteristics of 19 patients followed to D7

| Number | Outcome | Gender | Age | Microorganisms | Septic shock |
| --- | --- | --- | --- | --- | --- |
| 1 | S | F | 21 | Mycobacterium tuberculosis | N |
| 2 | S | F | 61 | Acinetobacter baumannii | N |
| 3 | S | F | 56 | Pseudomonas aeruginosa | N |
| 4 | S | M | 66 | Fungi | N |
| 5 | S | M | 67 | Pseudomonas aeruginosa | N |
| 6 | S | M | 71 | Stenotrophomonas maltophilia | N |
| 7 | S | F | 64 | Acinetobacter baumannii | Y |
| 8 | S | M | 78 | Acinetobacter baumannii | N |
| 9 | S | F | 70 | Bacillus levans | N |
| 10 | S | M | 55 | Pseudomonas aeruginosa | Y |
| 11 | NS | M | 79 | Acinetobacter baumannii | N |
| 12 | NS | M | 73 | Acinetobacter baumannii | Y |
| 13 | NS | M | 78 | Acinetobacter baumannii | Y |
| 14 | NS | F | 71 | Pseudomonas aeruginosa | N |
| 15 | NS | M | 34 | Staphylococcus aureus | Y |
| 16 | NS | M | 72 | Acinetobacter baumannii | N |
| 17 | NS | F | 79 | Fungi | N |
| 18 | NS | M | 42 | Virus | Y |
| 19 | NS | F | 53 | Mycobacterium tuberculosis | Y |

S=survivors; NS=nonsurvivors; F=female; M=male; Y=yes; N=no.
